# From underdiagnosis to effective management of self-harming behaviors. The role of trazodone in patients with intellectual disabilities affected by anxiety and depression

**DOI:** 10.1192/j.eurpsy.2026.11249

**Published:** 2026-07-31

**Authors:** O. Gori, M. Balestri, R. Raggini, S. Costantini, A. Fabbri, R. P. P. Sant’angelo

**Affiliations:** Mental Health Department, Ausl Romagna, Cesena, Italy

## Abstract

**Introduction:**

In literature, 15–20% of individuals with severe intellectual disabilities display non-suicidal self-injurious behaviors (SIB), rising to 30–50% in those with Autism Spectrum Disorder (ASD). The causes of SIB are complex, involving biological, psychological, and environmental factors. These behaviors can cause injuries, infections, and scarring, significantly impacting caregivers’ and healthcare professionals’ burden and reducing quality of life for all involved.

**Objectives:**

This report highlights two clinical cases illustrating the crucial role of accurate diagnosis and tailored treatment in managing SIB.

**Methods:**

The severity and persistence of SIB are often worsened by undiagnosed psychiatric comorbidities.

**Results:**

Dario, 28, has level 3 autism spectrum disorder and severe intellectual disability. Since age 7, he exhibits self-injurious behaviors (SIB), mainly hitting ears and head, causing bilateral cauliflower ear. Recently, SIB worsened, with biting, wounds, and aggression. During agitation, he was hospitalized and treated with anesthetics and high-dose neuroleptics, but without improvement; complications like dysphagia and pneumonia arose. Transferred to the Psychiatric Ward, SIB was linked to depression and anxiety due to environmental changes. Pharmacological therapy was reviewed considering its metabolism via cytochrome P450, with ultra-rapid CYP1A2 and slow CYP2D6 activity.

Margherita, 19, with moderate intellectual disability and a family history of psychiatric disorders and suicides, has worsened since September 2024, linked to stress. Despite multiple hospitalizations and therapy adjustments, she shows self-injury, agitation, irritability, aggressiveness, and sleep issues. The treatment prior to hospitalization, which included two off-label antipsychotics, proved ineffective and caused significant side effects such as sialorrhea, motor slowing, rigidity, and aspiration pneumonia. Within a few days, the patient was discharged with a new therapeutic plan.

In both cases, the antipsychotic dose was reduced and trazodone treatment started. Trazodone was initially given intravenously or orally in immediate-release form for its sedative effect, alongside once-daily evening dosing. There was a significant reduction in self-injurious, aggressive, and agitated behaviors, as measured by the Aberrant Behavior Checklist.

**Conclusions:**

Psychiatric disorders are often underdiagnosed in individuals with intellectual disabilities; symptoms and behaviors are frequently wrongly attributed to the disability rather than a psychiatric condition (diagnostic overshadowing). Recognizing and treating disorders such as anxiety and depression can lead to clinical improvements, including reduced aggressive behaviors.

Trazodone is effective and safe in treating anxiety and depressive disorder in individuals with intellectual disabilities.

**Disclosure of Interest:**

None Declared

